# A Novel C-Type Lysozyme from *Mytilus galloprovincialis*: Insight into Innate Immunity and Molecular Evolution of Invertebrate C-Type Lysozymes

**DOI:** 10.1371/journal.pone.0067469

**Published:** 2013-06-20

**Authors:** Qing Wang, Chunyan Wang, Changkao Mu, Huifeng Wu, Linbao Zhang, Jianmin Zhao

**Affiliations:** 1 Key Laboratory of Coastal Zone Environmental Processes and Ecological Remediation, Yantai Institute of Coastal Zone Research, Chinese Academy of Sciences, Yantai, People’s Republic of China; 2 School of Marine Science, Ningbo University, Ningbo, People’s Republic of China; NIAID, United States of America

## Abstract

A c-type lysozyme (named as MgCLYZ) gene was cloned from the mussel *Mytilus galloprovincialis*. Blast analysis indicated that MgCLYZ was a salivary c-type lysozyme which was mainly found in insects. The nucleotide sequence of MgCLYZ was predicted to encode a polypeptide of 154 amino acid residues with the signal peptide comprising the first 24 residues. The deduced mature peptide of MgCLYZ was of a calculated molecular weight of 14.4 kD and a theoretical isoelectric point (*p*I) of 8.08. Evolution analysis suggested that bivalve branch of the invertebrate c-type lysozymes phylogeny tree underwent positive selection during evolution. By quantitative real-time RT-PCR (qRT-PCR) analysis, MgCLYZ transcript was widely detected in all examined tissues and responded sensitively to bacterial challenge in hemocytes and hepatopancreas. The optimal temperature and pH of recombinant MgCLYZ (rMgCLYZ) were 20°C and 4, respectively. The rMgCLYZ displayed lytic activities against Gram-positive bacteria including *Micrococcus luteus* and *Staphyloccocus aureus*, and Gram-negative bacteria including *Vibrio anguillarum*, *Enterobacter cloacae*, *Pseudomonas putida*, *Proteus mirabilis* and *Bacillus aquimaris*. These results suggest that MgCLYZ perhaps play an important role in innate immunity of *M. galloprovincialis*, and invertebrate c-type lysozymes might be under positive selection in a species-specific manner during evolution for undergoing adaptation to different environment and diverse pathogens.

## Introduction

Invertebrates, which lack acquired immunity, are exclusively dependent on innate immunity [1]. Like other invertebrates, the innate immunity of bivalves consists of both cellular and humoral defenses [2]. The former includes phagocytosis or encapsulation of pathogens, with subsequent pathogen destruction via enzyme activity and oxygen metabolite release, while the latter includes various reactions mediated by series of molecules such as antimicrobial peptides and proteins [3], [4]. Among the large number of inducible antimicrobial molecules, lysozyme is one of the most ubiquitous antibacterial factors and is widely distributed in invertebrate animals [1], [5].

Lysozymes (EC 3.2.1.17) are enzymes that cleave β-1,4 glycosidic bond of peptidoglycan in bacterial cell wall, and have been identified in different organisms including animals, plants and bacteriophages [6]. Lysozymes identified in animals are generally classified into three types: chicken-type (c-type), goose-type (g-type) and invertebrate-type (i-type) [7]. It has been demonstrated that c-type lysozymes are widely found in vertebrates (mammals, birds, reptiles, fish) and in some invertebrates (insects, crustaceans) [8].

Invertebrate c-type lysozymes are predominately identified in Arthropoda, and numerous studies on c-type lysozymes have been reported in insects and crustaceans [8]. It has been demonstrated that invertebrate c-type lysozymes function mainly in innate immunity against the invasion of bacterial pathogens [9]–[10]. For example, c-type lysozymes from crustaceans [11]–[14] and insects [15]–[16] displayed bactericidal activity towards both Gram-positive and Gram-negative bacteria. Moreover, invertebrate c-type lysozyme may also have anti-virus activity, for injection of recombinant c-type lysozyme could protect blue shrimp from white spot syndrome virus (WSSV) infection [17].

Until recently, only two c-type lysozymes have been characterized from the clam *Cyclina sinensis* [18] and the abalone *Haliotis discus hannai* [19]. The antibacterial activity of abalone c-type lysozyme and its expression profile after bacterial stimulation have been studied. However, the function of c-type lysozyme from bivalve has not been reported so far. Additionally, the evolutionary mechanism of invertebrate c-type lysozymes was not well studied. In this study, a novel c-type lysozyme was characterized from *M. galloprovincialis*. The antibacterial activity, tissue-specific expression and temporal expression patterns post bacterial challenge were investigated. Besides, the evolution of invertebrate c-type lysozyme was also discussed.

## Materials and Methods

### Animals and Bacterial Challenge

The mussels (shell-length: 3.0–5.0 cm) were collected from a local mussel culturing farm and acclimatized in aerated seawater (32 psu, pH 8.0) at 20°C for 7 days before commencement of the experiment. No specific permits were required for the described field studies. The mussels were fed with a mixture of *Isochrysis galbana* and *Phaeodactylum tricornutum*, and the seawater was totally renewed daily.

After acclimatization, the mussels were randomly divided into three experimental groups with three replicates each containing 30 individuals. For the challenge group, 50 µL of live *V. anguillarum* (1×10^7^ CFU/mL) re-suspended in sterilized seawater was injected into the adductor muscle of each mussel. For the control group, the mussels were injected with an equal volume of sterilized seawater respectively. The untreated mussels were employed as the blank group. The hemocytes and hepatopancreas of six mussels (two mussels for each replicate) from each group were randomly collected for RNA extraction at 6, 12, 24, 48, 72 and 96 hours post the challenge. The hemocytes, hepatopancreas, gill, mantle, gonad and adductor muscle of six untreated mussels were also sampled for total RNA extraction.

### Total RNA Extraction, cDNA Synthesis and Cloning the Full-length cDNA

Total RNA was extracted according to the manufacture’s protocol (Invitrogen, USA) and cDNA synthesis was conducted as described previously [20]. One expressed sequence tag (EST) sequence homologous to previously identified c-type lysozyme was identified from a hemocyte cDNA library (unpublished). The 5′ and 3′ ends of MgCLYZ were obtained by rapid amplification of cDNA ends using the SMART RACE cDNA Amplification Kit (Clontech, USA) according to the manufacture’s protocols.

### Bioinformatics Analysis

The cDNA sequence and deduced amino acid sequence of MgCLYZ were analyzed using the BLAST algorithm (http://www.ncbi.nlm.nih.gov/blast) and the Expert Protein Analysis System (http://www.expasy.org/), respectively. The ClustalW program was used to conduct multiple alignments (http://www.ebi.ac.uk/clustalw/). Protein domain prediction was conducted using simple modular architecture research tool (SMART) software (http://smart.embl-heidelberg.de/). The signal peptide was predicted by SignalP 4.0 server (http://www.cbs.dtu.dk/services/SignalP/). The DiANNA server (http://clavius.bc.edu/~clotelab/DiANNA/) was utilized to calculate the disulfide connectivity. The secondary structure was predicted using the PSIPRED Server (http://bioinf.cs.ucl.ac.uk/psipred/) and the three-dimensional structure was predicted by SWISS-MODEL (http://swissmodel.expasy.org/workspace). A maximum likelihood (ML) phylogenetic tree based on the amino acid sequences was constructed using PhyML 3.0 [21]. ProtTest version 2.4 [22] was used to identify the best-fit model of amino acid substitution, and the WAG+I+G model was selected as the best using AIC. For ML analysis, 100 bootstraps were used to estimate the node reliability. The sequences used for multiple alignments and phylogenetic and evolution analysis were listed in Table 1.

**Table 1:** Sequences used for multiple alignment, phylogenetic and evolution analysis.

lysozyme type	Species	Accession numbers	Taxonomy	Sequence size
c-type lysozyme	*Mytilus galloprovincialis*	AFM43653.1	Bivalvia	154aa
lysozyme	*Cyclina sinensis*	AEG19518.1	Bivalvia	155aa
c-type lysozyme	*Haliotis discus hannai*	ADR70995.1	Gastropoda	146aa
salivary lysozyme	*Simulium nigrimanum*	ACZ28238.1	Insecta	141aa
salivary lysozyme	*Simulium vittatum*	ACH56920.1	Insecta	141aa
lysozyme	*Anopheles gambiae*	AAC47326.1	Insecta	140aa
salivary lysozyme	*Anopheles stephensi*	AAO74844.1	Insecta	141aa
lysozyme	*Manduca sexta*	AAB31190.2	Insecta	138aa
lysozyme	*Musca domestica*	ACE00424.1	Insecta	141aa
lysozyme	*Pseudoplusia includens*	AAS48094.1	Insecta	141aa
lysozyme	*Heliothis virescens*	AAD00078.1	Insecta	141aa
lysozyme	*Helicoverpa armigera*	ABF51015.1	Insecta	135aa
lysozyme	*Ostrinia nubilalis*	ADU33188.1	Insecta	140aa
lysozyme	*Spodoptera litura*	ACI16106.1	Insecta	141aa
lysozyme P	*Drosophila melanogaster*	NP_476828.1	Insecta	141aa
c-type lysozyme	*Schistocerca gregaria*	AEX60965.1	Insecta	141aa
c-2 lysozyme	*Periplaneta americana*	AFI81523.1	Insecta	139aa
lysozyme	*Culex tarsalis*	ACJ64375.1	Insecta	148aa
lysozyme	*Dermacentor variabilis*	AAO23571.1	Arachnida	139aa
lysozyme	*Haemaphysalis longicornis*	BAK20441.1	Arachnida	140aa
c-type lysozyme	*Marsupenaeus japonicus*	BAC57467.1	Crustacea	158aa
lysozyme	*Fenneropenaeus chinensis*	AAV83994.1	Crustacea	158aa
lysozyme	*Fenneropenaeus indicus*	ACV49870.1	Crustacea	158aa
lysozyme	*Litopenaeus vannamei*	AAL23948.1	Crustacea	158aa
lysozyme	*Macrobrachium rosenbergii*	AAP13577.2	Crustacea	158aa
lysozyme	*Penaeus monodon*	ABU75288.1	Crustacea	158aa
lysozyme	*Scylla paramamosain*	ADM33942.1	Crustacea	223aa
lysozyme	*Portunus trituberculatus*	ACM24796.2	Crustacea	223aa
c-type lysozyme	*Scophthalmus rhombus*	BAF75844.1	Teleostei	143aa
lysozyme c	*Bos taurus*	DAA29810.1	Mammalia	147aa
lysozyme precursor	*Homo sapiens*	AAA36188.1	Mammalia	148aa

### Testing for Positive Selection

The open reading frame (ORF) nucleotide sequences encoding amino acids of c-type lysozymes from invertebrates were used to construct the neighbor-joining (NJ) phylogeny tree with Kimura 2-parameter model. The reliability of interior branches of each phylogeny was assessed with 1000 bootstraps. The phylogeny was used to estimate non-synonymous to synonymous rate ratio (ω = dN/dS) by CODEML program of the PAML 4.4 software package [23]. Values of ω >1 indicate positive selection, while ω = 1 and ω <1 indicate neutral evolution and purifying selection. Likelihood ratio tests (LRTs) were used to determine whether any codon positions were subjected to positive selection.

The free-ratio model, which assumes a different ω parameter for each branch in the phylogenetic tree, is applied to test for detecting positive selection acting on particular lineages. The site-specific models M1a (nearly neutral)/M2a (positive selection), M7 (beta)/M8 (beta & ω) were used to test for selective pressure at amino acid sites. The Naive Empirical Bayes (NEB) method and Bayes empirical Bayes (BEB) method were used to calculate the posterior probabilities that each codon is from the site class of positive selection under models M2a and M8 respectively [24].

### Tissue Distribution and Expression Profiles Post *V. anguillarum* Challenge

Quantitative real time RT-PCR (qRT-PCR) was carried out by using an ABI 7500 Real-time Detection System with the SYBR ExScript qRT-PCR Kit (Takara, China) as described previously [25]. The comparative CT method (2^−ΔΔCT^ method) was used to analysis the expression level of MgCLYZ [26]. The β-actin gene of *M. galloprovincialis* was used as a reference gene [25], [27]. The primers used to quantify the relative expression level of MgCLYZ were listed in Table 2.

**Table 2:** Primers used in this study.

Primer	Sequence (5′–3′)	Sequence information
P1 (forward)	TGTAACAAACTGGGACGATA	Real time primer for β-actin
P2 (reverse)	AGCATGAGGAAGGGCATAAC	Real time primer for β-actin
P3 (forward)	GCACCTCATTGACTAACTCGG	Real time primer for MgCLYZ
P4 (reverse)	CTGAACCCTGGACATTGGAAC	Real time primer for MgCLYZ
P5 (forward)	CCATGGGCTACAAAAACGAAGTGCCA	Recombinant primer for MgCLYZ
P6 (reverse)	CTCGAGTTAGTGGTGGTGGTGGTGGTGACATGTGCTGTAATCGT	Recombinant primer for MgCLYZ

The data were analyzed by one-way analysis of variance (one-way ANOVA, Duncan's post-hoc test) using SPSS 16.0 software (SPSS Inc., USA). The *P* values less than 0.05 were considered statistically significant.

### Recombinant Expression and Mass Spectrometric Identification

PCR fragment encoding the mature peptide of MgCLYZ was amplified with two gene-specific primers (Table 2) with *Nco* I and *Xho* I sites, respectively. The PCR product was cloned into pMD18-T simple vector (Takara, China), digested completely by restriction enzymes *Nco* I and *Xho* I (NEB), and then subcloned into the *Nco* I/*Xho* I sites of expression vector pET-21a(+) (Novagen, Germany). The recombinant plasmid (pET-21a-MgCLYZ) was transformed into *Escherichia coli* BL21 pLysS (DE3) (Novagen, Germany). The recombinant proteins were expressed as inclusion bodies and purified by HisTrap Chelating Columns (GE Healthcare, USA) under denatured condition (8 mol/L urea). The purified rMgCLYZ was analyzed by SDS-PAGE with separation in a 15% gel followed by Coomassie brilliant blue R250 (CBB-R250) staining.

After SDS-PAGE, the target protein band was excised from the gel and cut into small pieces. The gels were processed and digested according to the method described by Katayama et al [28]. The gels were washed three times with ultrapure water and decolorized with 25 mmol/L NH_4_HCO_3_ (in 50% v/v acetonitrile) at room temperature for 30 min. After being dried in 50% acetonitrile for 30 min and 100% acetonitrile for another 30 min, the samples were rehydrated in 10 µL cover solution (0.02 g L^−1^ w/v trypsin, 25 mM NH_4_HCO_3_ and 10% acetonitrile) for 30 min, and then covered with the same solution but without trypsin for digestion overnight at 37°C. The supernatants were extracted with 5% trifluoroacetic acid (TFA) in 67% acetonitrile at 37°C for 30 min, and then centrifuged at 5 000 *g* for 5 min. The samples which were completely dried were re-suspended with 5 µL 0.1% TFA followed by mixing in 1∶1 ratio with a saturated solution of α-cyano-4-hydroxy-trans-cinnamic acid in 50% acetonitrile. One microliter of the mixture was analyzed by an ABI 4800 MALDI-TOF/TOF Plus mass spectrometer (Applied Biosystems, USA). Both the MS and MS/MS data were integrated and processed using the GPS Explorer V3.6 software (Applied Biosystems, USA) with default parameters.

### Lysozyme Activity Assay

The purified rMgCLYZ was refolded in gradient urea-TBS glycerol buffer according to the method described by Yang et al [29]. The concentration of purified recombinant protein was measured by BCA method [30]. The optimal pH, temperature of MgCLYZ activity was determined using *Micrococcus lysodeikticus* as the substrate with the method described by Xue et al [31]. The pHs ranging from 2 to 10, and the temperatures ranging from 10°C to 60°C were used. The measurements of lysozyme activity were done in triplicate and the results were shown as percent activity with the highest activity being defined as 100%.

### Antibacterial Activity of rMgCLYZ

Two Gram-positive bacteria (Micrococcus luteus, Staphyloccocus aureus) and five Gram-negative bacteria (Vibrio anguillarum, Enterobacter cloacae, Pseudomonas putida, Proteus mirabilis, Bacillus aquimaris) were used in antibacterial test. The minimum inhibitory concentration (MIC) was determined according to the method of Hancock (http://cmdr.ubc.ca/bobh/methods/) as described previously [25]. The plates with V. anguillarum and P. putida were incubated at 28°C for 24 h, and the plates with other tested bacteria were incubated at 37°C for 24 hours.

## Results

### Sequence Analysis of MgCLYZ cDNA

**Figure 1:**
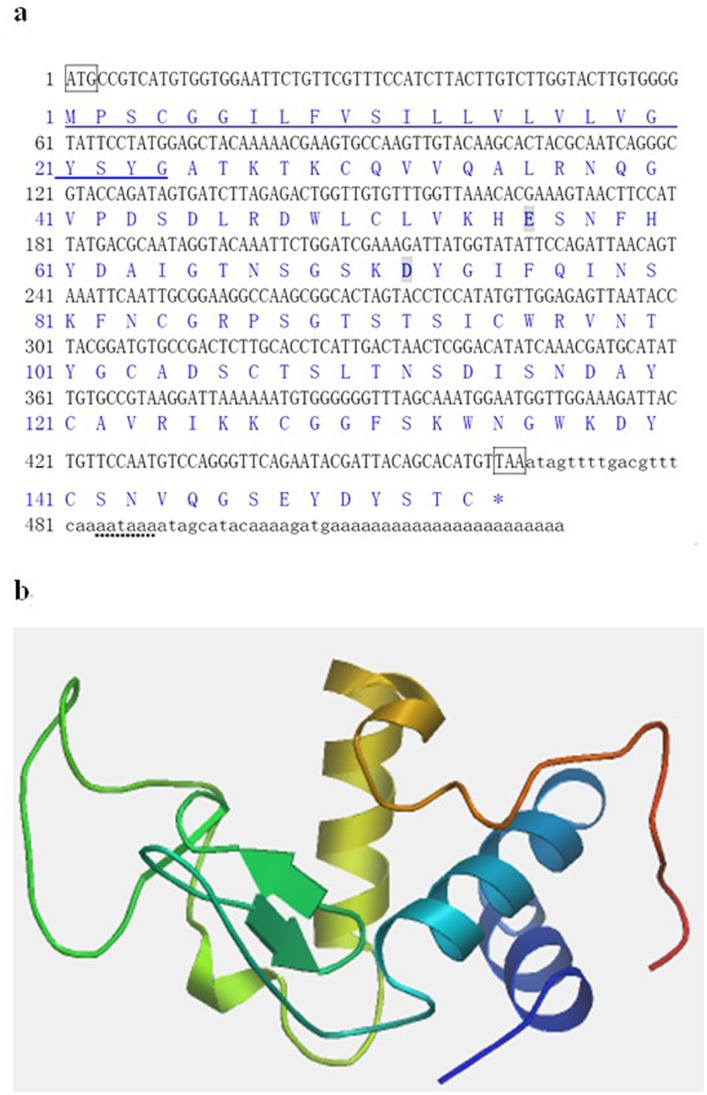
Nucleotide sequence (a) and three-dimensional structure (b) of MgCLYZ. The asterisk (*) indicates the stop codon. The start and stop codons are included in a box. The signal peptide and polyadenylation signal are underlined with solid line and dot line, respectively. The active catalytic residues are in bold and shaded in dark.

The full-length cDNA sequence of MgCLYZ was deposited in GenBank under the accession no. JQ863366. The MgCLYZ sequence had an ORF of 462 bp encoding a polypeptide of 154 amino acids with the signal peptide comprising the first 24 residues (Figure 1a). The deduced mature protein had a predicted molecular mass of 14.4 kDa and a theoretical pI of 8.08. SMART program analysis revealed that MgCLYZ contained a lysozyme C/Alpha-lactalbumin domain (residues 25–150). The mature peptide of MgCLYZ possesses 10 Cys residues, of which, Cys ^51^ and Cys ^141^, Cys^ 84^ and Cys ^107^, Cys ^103^ and Cys ^121^, and Cys ^128^ and Cys ^154^ were predicted to form disulfide bonds. As predicted by PSIPRED program, MgCLYZ had a secondary structure with three α-helixes and two β-sheets. The predicted three-dimensional structure of MgCLYZ was divided into two domains by a deep cleft containing the active site. One domain mainly consisted of the β-sheet structure, while the other domain was more helical in nature (Figure 1b).

### Multiple Sequences Alignment and Phylogenetic Analysis

**Figure 2:**
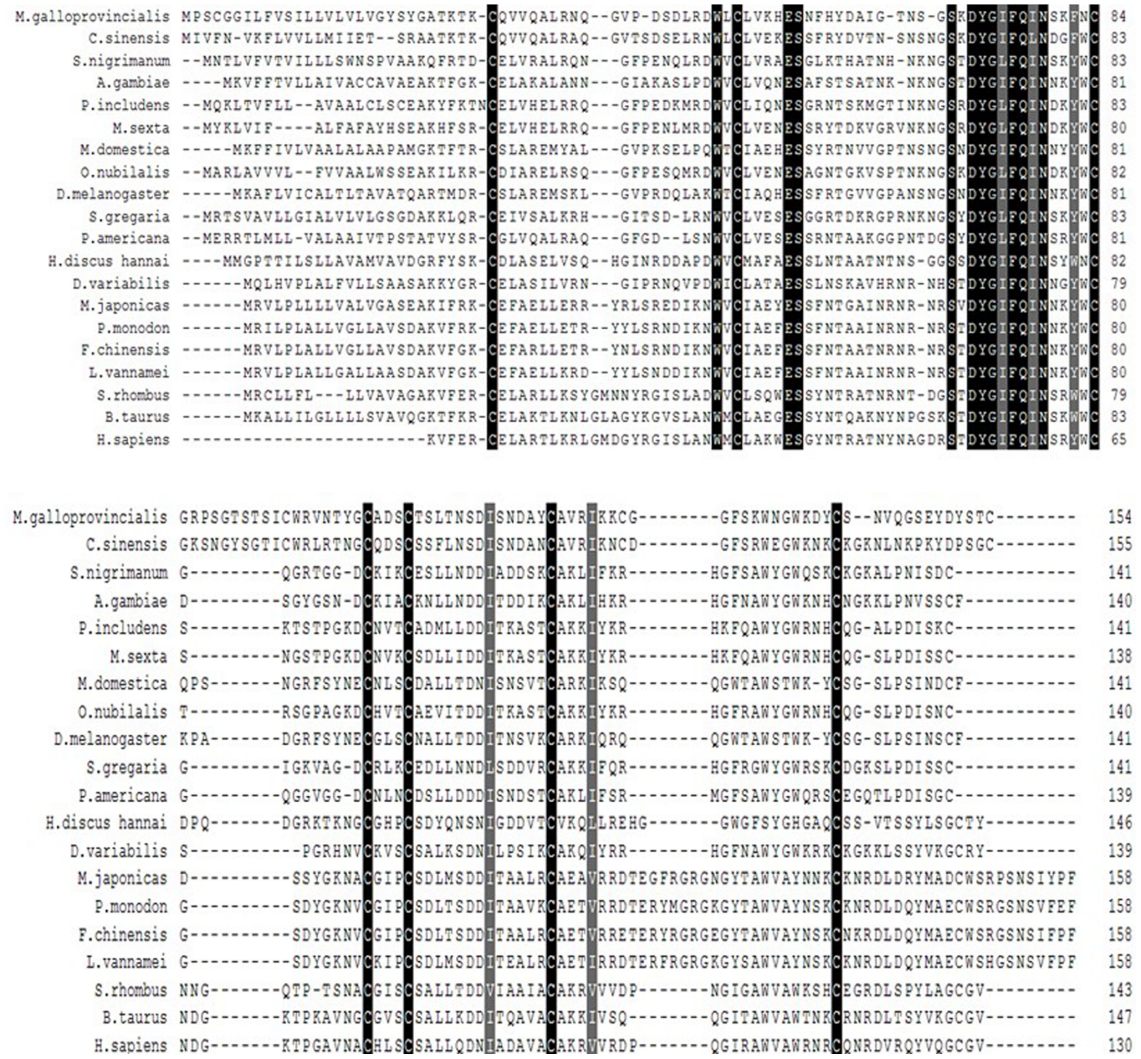
Multiple alignments of MgCLYZ with other c-type lysozymes orthologs deposited in GenBank. The black shadow region indicates positions where all sequences share the same amino acid residue. Gaps are indicated by dashes to improve the alignment. The GenBank accession numbers and the species are shown in Table 1.

Blast analysis revealed that MgCLYZ shared a high homology with lysozymes from the bivalve *Cyclina sinensis* (AEG19518.1, 64% identity), the mosquito *Anopheles stephensi* (AAO74844.1, 45% identity) and the insect *Simulium nigrimanum* (ACZ28238.1, 45% identity). The deduced amino acid sequence of MgCLYZ was aligned with other known c-type lysozymes and several conserved features were found in MgCLYZ (Figure 2). The two catalytic residues (Glu ^56^ and Asp ^72^) and seven Cys residues were conserved in MgCLYZ, and the motif -DYGI(L)FQINS(N/D)R(K)Y(W)WC- was also conserved among species [32]. In addition, a short insertion sequences were observed in the bivalve lysozyme amino acid sequences. The sequence similarity and the common structure features suggested that MgCLYZ was a new counterpart of invertebrate c-type lysozymes.

**Figure 3:**
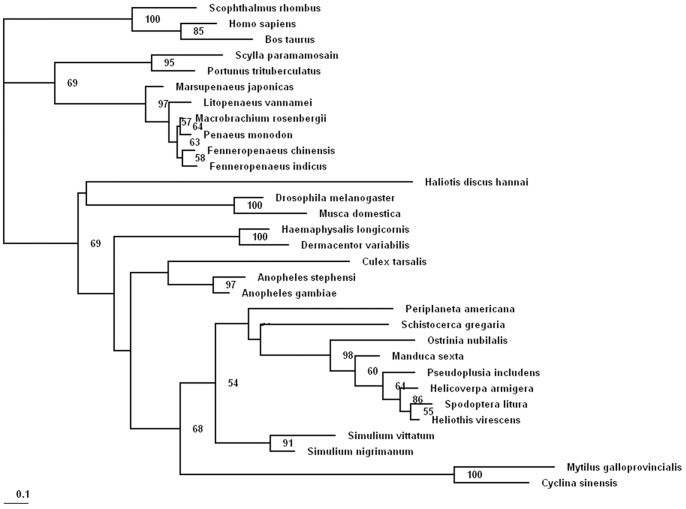
Phylogenetic tree constructed by maximum likelihood method based on the amino acid sequences of c-type lysozymes from invertebrate animals. Numbers at the forks indicate the bootstrap values (in %) out of 100 replicates. The sequences of c-type lysozymes from vertebrate animals (*Scophthalmus rhombus, Bos Taurus, Homo sapiens*) were used as an out-group. Bootstrap value <50 are not shown. The sequences used to construct phylogeny trees of c-type lysozymes are shown in Table 1.

The phylogenetic tree of invertebrate c-type lysozyme (Figure 3) included two major clades: c-type lysozymes from insects and mollusks, and c-type lysozymes from arthropods (c-type lysozymes from vertebrates as an out-group). MgCLYZ was firstly clustered with c-type lysozyme from the bivalve *C. sinensis*, and then grouped with c-type lysozymes mainly from lepidoptera and diptera insects, further grouped with c-type lysozyme of *Haliotis discus hannai*.

### Evolution Analysis of Invertebrate c-type Lysozymes

**Figure 4:**
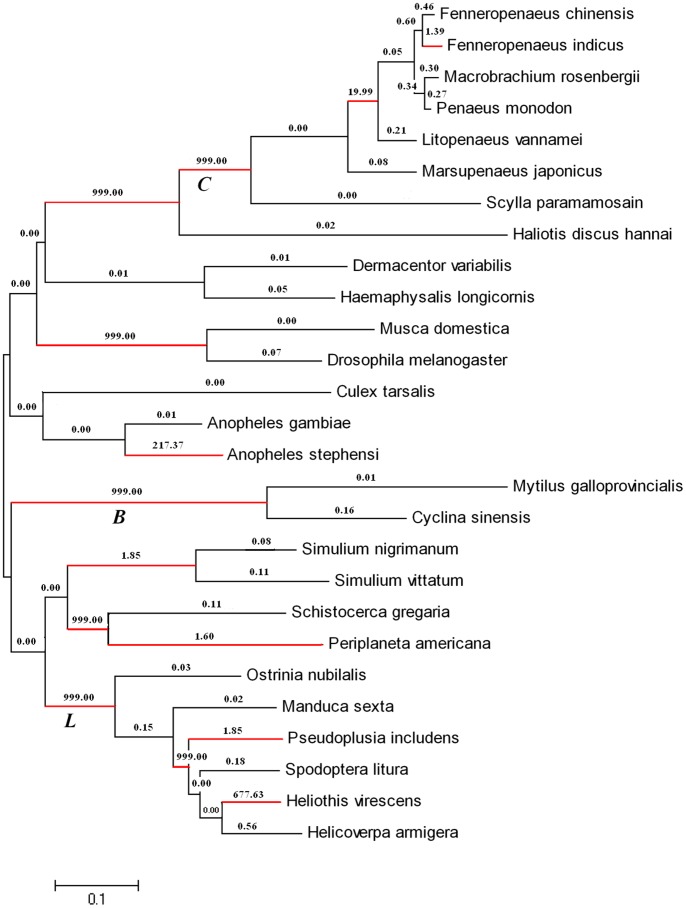
Positive selection at c-type lysozyme across the invertebrate phylogeny. The number shown along each branch is the non-synonymous to synonymous rate ratio for the entire gene along that branch. The branches in red show strong evidence of undergoing positive selection. *C* – Crustacea branch, *B* – Bivalvia branch, *L* – Lepidoptera branch.

The log-likelihood values and parameter estimates of c-type lysozyme from invertebrates under branch models and various site models were shown in Table 3. The free-ratio model was used, which assumed a different dN/dS ratio (parameter ω) for each branch in the tree. The log likelihood value under this model was *l*_1_ = −8505.0. The one-ratio model, which assumed the same ω parameter for the entire tree, led to *l*_0_ = −8552.0. The M1 model was compared with model M0 to determine the existence of ω heterogeneity among the branches in the phylogeny. The M1–M0 comparison revealed that M1 was better fit to the data (2Δ*l* = 94, χ^2^ = 86.66, df = 50, *P*<0.001), indicating the ω ratios were indeed different among lineages. The lineage-specific selection test showed that the ω values along the most (36 out of 51) examined lineages were less than 1. However, the ω values of many branches including some internal branches were more than 1, evidencing their evolution under positive selection (Figure 4). Maximum-likelihood estimates of parameters indicated that 71.5 nonsynonymous and 0 synonymous substitutions occurred along Bivalvia (*B*) branch (Figure 4), suggesting that the bivalve lineage undergoes positive selection. However, the ω ratio value for *M. galloprovincialis* clade was very low, implying no positive selection on this lineage. The Crustacea (*C*) branch and Lepidoptera (*L*) branch also had very high ω ratios (Figure 4), revealing that these c-type lysozymes are under positive selection pressure during evolution. In addition, there were also some branches which consisted of c-type lysozymes from different taxon with high ω ratios.

**Table 3:** Parameter estimates and log-likelihood values under different models of variable ω ratios among sites. Site numbers and amino acids refer to the *M. galloprovincialis* sequence.

Model	Model code	lnL	Estimates of parameters	2Δ*l*	*P* value	Positively selected sites
branch model	One ratio model	−8552.0	ω = 0.06120	94 (df = 50)	*P*<0.001	NA
	Free-ratio model	−8505.0	ω estimated independently for each branch(see Fig. 4)			
site model	M1a	−8949.5	P0 = 0.00001 (p1 = 0.99999)	0	*P*>0.05	Not allowed
	M2a	−8949.5	p0 = 0.00000, p1 = 1.0000, (p2 = 0.00000), ω0 = 0.00000, (ω1 = 1), ω2 = 1.00000			4T, 21D, 77Y
	M7	−8502.6	p = 2.08566, q = 28.78652	5.2	*P*>0.05	Not allowed
	M8	−8500.0	p0 = 0.99031, p = 2.21027, q = 32.22614, (p1 = 0.00969), w = 1.00000			None

The site-specific models were used to test for heterogeneous selective pressure at amino acid sites. The M1a–M2a comparison revealed that M1a was better fit to the data (2Δ*l* = 0, χ^2^ = 5.99, df = 2, *P*>0.05). LRTs also gave significantly better results for M7 (2Δ*l* = 5.2, χ^2^ = 5.99, df = 2, *P*>0.05). These results suggested that all the amino acids were under nearly neutral selection detected by these models.

### Tissue Distribution and Temporal Expression Profiles Post Bacterial Challenge

**Figure 5:**
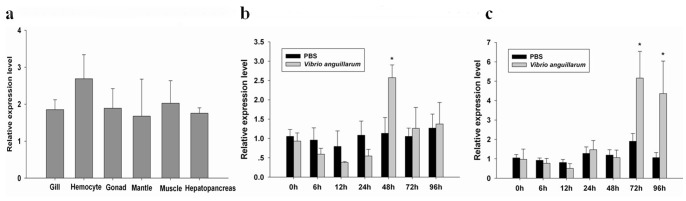
Tissue-specific expression (a) and temporal expression profiles post bacterial challenge in hemocytes (b) and hepatopancreas (c). The mRNA expression level is calculated relative to actin expression and shown as mean ± SE (n = 6). Significant difference from control is indicated with an asterisk at *P*<0.05.

By qRT-PCR analysis, the MgCLYZ transcripts were detected in all the tissues examined, including hepatopancreas, hemocytes, muscle, mantle and gills (Figure 5a). No significant difference in the expression of MgCLYZ mRNA was observed in the examined tissues, indicating a wide tissue expression of MgCLYZ transcript.

The expression level of MgCLYZ transcript in hemocytes decreased at the first 12 h post challenge but then increased at the following times. The expression level reached a maximum value of 2.57-fold at 48 h post challenge, which was significantly higher than that of the control (*P*<0.05) (Figure 5b). In hepatopancreas (Figure 5c), the expression level of MgCLYZ mRNA did not change until 48 h following the challenge, but then was up-regulated significantly at 72 h and 96 h post challenge compared with the control (*P*<0.05).

### Protein Recombinant and Mass Spectrometry Identification

**Figure 6:**
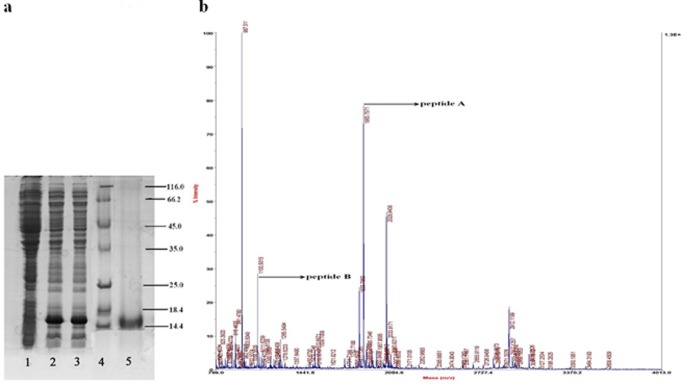
Analysis of recombinant MgCLYZ protein. (a) SDS-PAGE analysis of rMgCLYZ. About 5 microgram recombinant protein was loaded and the 15% gel was visualized by Coomassie brilliant blue R250 staining after electrophoresis. Lane 1: negative control for rMgCLYZ (without induction); Lane 2: induced expression for 4 h of rMgCLYZ; Lane 3: induced expression for 5 h of rMgCLYZ; Lane 4: protein molecular standard; Lane 5: purified rMgCLYZ. (b) MALDI-TOF/TOF identification of the peptide (peptide A: –HESNFHYDAIGTNSGSK–; peptide B: –NQGVPDSDLR–) of rMgCLYZ.

The rMgCLYZ was detected by SDS-PAGE and visualized as a protein of about 14 kDa (Figure 6a, Lane 5). One peptide fragment (–NQGVPDSDLR–) identified by MALDI-TOF/TOF was identical to 14–23 of the mature peptides of MgCLYZ (Figure 6b), and the other peptide fragment (–HESNFHYDAIGTNSGSK–) matched 31–47 of the mature peptides of MgCLYZ (Figure 6b).

### Lysozyme Activity of rMgCLYZ

**Figure 7:**
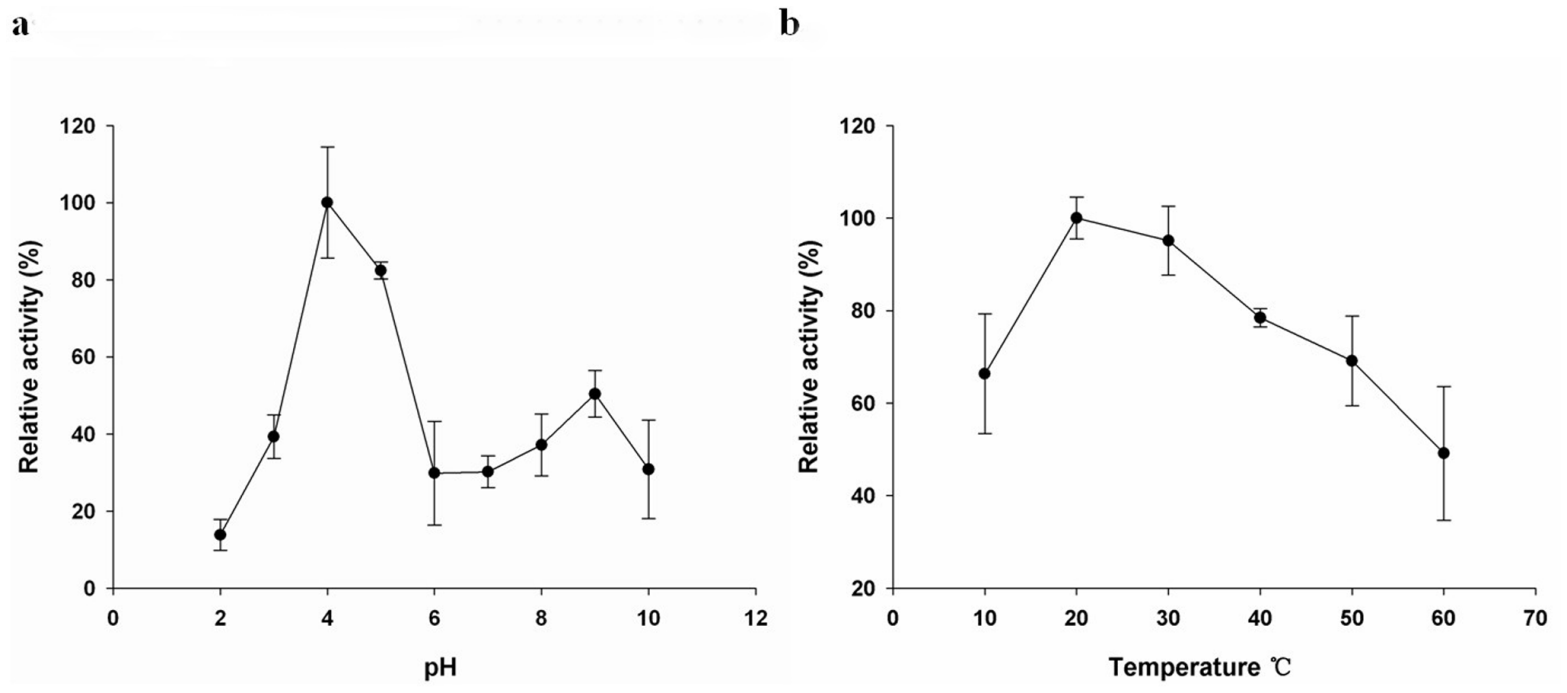
Effects of pH (a) and temperature (b) on the activity of rMgCLYZ. Lysozyme activity is shown as % of the highest activity. The values were shown as mean ± SE (n = 3).

The lytic activities of rMgCLYZ were detected at pHs ranging from 2 to 10 (Figure 7a). The rMgCLYZ had the highest activity at pH 4 and a relatively high activity at pH 5. The lowest activity was detected at pH 2, which was only about 14 percent of the highest activity. The high lytic activity of rMgCLYZ was found at temperatures ranging from 20 to 40°C and the optimal temperature for rMgCLYZ lysozyme activity was 20°C (Figure 7b). At the temperature above 20°C, the activity of rMgCLYZ decreased with the increasing temperatures and reached to the minimum value at 60°C.

### Antibacterial Activity of rMgCLYZ

The spectrum of antibacterial activity of rMgCLYZ was investigated against several Gram-positive and Gram-negative bacteria (Table 4). The rMgCLYZ possessed a relatively strong lytic activity against *M. luteus*, *V. anguillarum* and *P. putida*, while it had a weak antibacterial activity against *S. aureus*, *E. cloacae, P. mirabilis* and *B. aquimaris*. The highest activity of MgCLYZ was found against *M. luteus* with a MIC of 1.76–3.51 µM. Thus, rMgCLYZ might be a broad-spectrum antibacterial protein.

**Table 4:** MIC values of recombinant MgCLYZ.

Tested microorganisms	MIC values
Gram-positive bacteria	
*Micrococcus luteus*	1.76–3.51 µM
*Staphylococcus aureus*	7.02–14.05 µM
Gram-negative bacteria	
*Vibrio anguillarum*	3.51–7.02 µM
*Bacillus aquimaris*	7.02–14.05 µM
*Enterobacter cloacae*	7.02–14.05 µM
*Pseudomonas putida*	3.51–7.02 µM
*Proteus mirabilis*	7.02–14.05 µM

## Discussion

In the present study, the cDNA sequence of a novel c-type lysozyme was characterized from mussel *M. galloprovincialis*. MgCLYZ possesses a typical c-type lysozyme domain containing the conserved residues (Glu ^56^ and Asp ^72^) essential to catalytic activity [33]. Unlike eight conserved Cys residues in most c-type lysozymes, only seven Cys residues were conserved in MgCLYZ. However, four disulfide bonds were predicted to form in MgCLYZ, which were critical to the maintenance of structural stability [7]. In addition, MgCLYZ contains an Asn ^83^ residue in a position where there should be a conserved Trp residue in the signature motif of “-DYGI(L)FQINS(N/D)R(K)Y(W)WC-”. It has been proposed that the conserved Trp residue in chicken and human c-type lysozyme is involved in substrate binding. The change of this residue may influence the binding specificity and efficiency of MgCLYZ toward the substrates [34]–[35]. The c-type lysozymes are classified into two different subfamilies, including the calcium-binding and conventional non-calcium-binding families [36]. Due to the absence of calcium binding Asp residues at positions 112, 117 and 118 [37], MgCLYZ was suggested as a new member of the non-calcium binding family.

Compared to vertebrate and arthropoda c-type lysozymes, mollusk counterparts have not been well characterized. Presently, only one c-type lysozyme from gastropod and two c-type lysozymes (including MgCLYZ) from bivalve have been reported [18]–[19]. In addition, we have characterized two c-type lysozymes in manila clam *Ruditapes philippinarum* recently (data not shown). Therefore, the bivalves may have the same diversity of c-type lysozyme as other lineages. However, analysis of complete genome sequences available in NCBI showed that c-type lysozyme was absent from *Crassostrea gigas*, suggesting that lineage-specific gene loss might occur during the evolution. In addition, c-type lysozyme are also absent from the phylum of Echinodermata and Urochordata [7]. In the Mollusca phylum, i-type and g-type lysozymes have been reported previously. Consequently, all three types of animal lysozymes had been found to coexist in mollusks [8]. Our study suggested that *M. galloprovincilias* was the first species in which i-, g- and c-type lysozymes coexist.

The phylogenetic analysis indicated that the bivalve c-type lysozymes were closer evolutionarily to c-type lysozymes from insects than gastropod and crustaceans. This might be explained that the c-type lysozymes from different invertebrate phylum have evolved under different selection pressures to adapt different environments and diverse pathogens. Therefore, the phylogenetic relationship of mussel and abalone based on c-type lysozymes was not quite consistent with traditional taxonomic classification.

Studies have shown that host genes involved in immunity are frequently subjected to positive selection during evolution [38]. The typical examples include major histocompatibility complex (MHC) class I [39] and α-defensin [40]. In the present study, although the ω values along the most (36 out of 51) examined lineages were less than 1, suggesting a common effect of functional constraint, the bivalve, lepidopter and crustacean lineages had very high ω values indicating that these lineages undergo positive selection during evolution. This result suggested that the c-type lysozymes might have been subjected to the positive selection since mollusk and this selection was remarkably variable among lineages. The heterogeneity in the evolutionary rate of invertebrate c-type lysozymes indicated that c-type lysozyme might undergo ecological adaptation in a complex species-specific manner [41]. It has been reported that the digestive c-type lysozymes from ruminants had evolved under positive selection pressure and six residues were found to undergo adaptive changes for digestive function [42]. However, our study showed that the bivalve c-type lysozymes perhaps underwent positive selection although no positive selected sites were detected.

In invertebrate animals, the tissue-distribution profiles of c-type lysozymes are species-dependent. It had been reported that shrimp c-type lysozyme transcripts were mainly expressed in immune organs or organs exposed to external environment, such as hemocytes and gills [10]–[11], [13]–[14], [17], [43]. In insects, many c-type lysozymes which were also called salivary lysozymes were mainly expressed in salivary glands of fruit fly, mosquitoes, termite and moth larvae [44]–[48]. Some c-type lysozymes were also highly expressed in midgut and fat body of mosquito and housefly [46], [49]. In abalone, the transcript of c-type lysozyme was found to be mainly expressed in mantle [19]. In contrast, MgCLYZ mRNA was widely expressed in all the examined tissues. This result suggested MgCLYZ might serve as a universal hydrolase against the bacteria in these tested tissues.

Expression profiles of c-type lysozymes post bacterial challenge had been investigated in many invertebrate animals. In this study, the expression level of MgCLYZ was significantly increased in hemocytes and hepatopancreas post bacterial challenge, indicating a role for MgCLYZ in the innate immune system of the mussel. Similar results have also been found in abalone, shrimp and mosquito [19], [43], [46], [50]. For example, the transcripts of c-type lysozymes were up-regulated post *M. luteus* and *V. alginolyticus* challenge in shrimps [43], [50]. In the mosquito *Anopheles gambiae*, lysozyme c-1 and c-2 transcripts increased significantly at 6–12 h post challenge with bacteria [46]. These results suggest that c-type lysozymes are immune responsive and involved in the immune responses of the invertebrates.

Previous studies showed that most avian and insect c-type lysozymes had antibacterial activities only against Gram-positive bacteria [36], [51]. However, some insect lysozymes also possessed lytic activities against Gram-negative bacterium *Escherichia coli* [52]–[53]. Recently, several shrimp c-type lysozymes demonstrated strong lytic activities against both Gram-positive and Gram-negative bacteria, especially several *Vibrio* species such as *V. alginolyticus* and *V. parahemolyticus* [11], [13]–[14], [17]. In abalone, the recombinant c-type lysozyme also showed bacteriolytic activity against both Gram-positive and Gram-negative bacteria [19]. In this study, MgCLYZ was found to display lytic activities against several Gram-positive and Gram-negative bacteria, providing the evidence of the involvement of c-type lysozyme in mussel immunity. Our results support the idea that marine invertebrate lysozymes have a wider range of activities than those of terrestrial invertebrates in order to cope with a great range of bacterial strains and species in the marine environment [11].

The optimal pH for the activity of mammal and chicken c-type lysozymes was generally ranging from 7 to 10 [7]. In crustacean, the recombinant tiger shrimp and kuruma shrimp lysozyme had optimum activity at pH 6.0 and 7.5, respectively [11], [13]. In the present study, it was found that rMgCLYZ had high activities within a narrow acidic pH ranging from 4 to 5. Similar results were also found in some ruminant and fish c-type lysozymes [42], [54]–[55]. Furthermore, rMgCLYZ exhibited high activities in the range of 10–30°C, which demonstrated that mussel c-type lysozyme was a low-temperature active enzyme. Similarly, some c-type lysozymes from marine shrimp and fish also had optimal temperatures in the range of 20–40°C [13], [56]. In marine environment where the temperature usually ranged from 0 to 30°C, so MgCLYZ could adapt to function in this temperature range [25]. Our findings are in agreement with previous deduction that cold-blooded aquatic animals possess cold-active lysozymes for protection from bacterial invasion in a wide range of environmental circumstances [11].

### Conclusions

In conclusion, a novel c-type lysozyme was characterized from *M. galloprovincialis*. The expression profiles post bacterial challenge, enzymatic property and antibacterial spectrum analysis suggested that MgCLYZ was involved in the host defense reaction. Further analysis indicated that the evolution of bivalve c-type lysozyme was obviously under positive selection. Our findings showed for the first time that c-type lysozyme played important roles in the innate immunity of *M. galloprovincialis*, and invertebrate c-type lysozymes probably had evolved under positive selection in a species-specific manner.
